# Utilizing the surface potential of a solid electrolyte region as the potential reference in Kelvin probe force microscopy

**DOI:** 10.3762/bjnano.13.129

**Published:** 2022-12-19

**Authors:** Nobuyuki Ishida

**Affiliations:** 1 National Institute for Materials Science, Sengen 1-2-1, Tsukuba, Ibaraki 305-0047, Japan

**Keywords:** electrochemistry, Kelvin probe force microscopy (KPFM), reference electrode, solid electrolyte

## Abstract

In electrochemical measurements, monitoring the electrode potential using a stable reference is essential for controlling the redox reactions that occur at the electrodes. In Kelvin probe force microscopy (KPFM) measurements on electrochemical cells, the surface potential is generally measured relative to electrical ground instead of a stable reference. Here, we show that the changes in the surface potential, measured using KPFM relative to the surface potential in the electrolyte region, is consistent with the changes in the electrode potential measured using a voltmeter relative to a reference electrode. These results demonstrate that the surface potential in the electrolyte region can be utilized as a stable potential reference when analyzing KPFM data.

## Introduction

Kelvin probe force microscopy (KPFM) is a scanning probe technique for imaging surface potentials on the nanometer scale [1–4]. Its operating principle is based on detecting the contact potential difference (CPD) between a tip and the sample. It has been used to evaluate a wide range of electronic and ionic devices [5–11]. The CPD is quantified by applying a regulated DC voltage, relative to an electrical ground, to the tip or the sample, to minimize the electrostatic force acting between the tip and sample [3]. CPD measurements relative to ground are not particularly problematic when analyzing KPFM data obtained from electronic devices [7,10–12] because the electrode potential relative to ground determines working conditions of the devices. In contrast, in the case of electrochemical devices such as batteries, the redox reactions that occur at the electrode are determined by the potential difference across the electrode–electrolyte interface, not the electrode potential relative to ground. This prevents the accurate consideration of redox reactions that occur at the electrode only from the CPD measured relative to ground [8–9]. To address this issue, conventional electrochemical measurements use a stable reference electrode as a third electrode to precisely measure the changes in the potential difference across the electrode–electrolyte interface [13]. In the case of KPFM measurements, the CPD can be measured not only at the electrodes but also over the electrolyte region. Therefore, the change in the potential difference across the electrode–electrolyte interface can, in principle, be detected without a reference electrode, although this has not yet been directly confirmed.

This study explored the possibility of obtaining a stable reference for KPFM measurements performed on electrochemical devices without a reference electrode. To this end, we prepared an electrochemical cell consisting of two Au electrodes and one Li reference electrode placed on a solid electrolyte (Li-ion conductor) substrate. The surface-potential distribution in the region across the solid electrolyte was measured with a DC voltage applied between the Au electrodes. During the KPFM measurements, the potential of each Au electrodes relative to the Li electrode was monitored using a voltmeter. Our analysis showed that the changes in the surface potential at each Au electrode, measured relative to the surface potential in the solid electrolyte region, agreed well with the changes in the Au electrode potential monitored by the voltmeter. This finding demonstrates that the potential in the solid electrolyte region can be utilized as a stable reference when analyzing KPFM data derived from samples for which attaching a reference electrode is difficult.

## Results and Discussion

**Figure 1:**
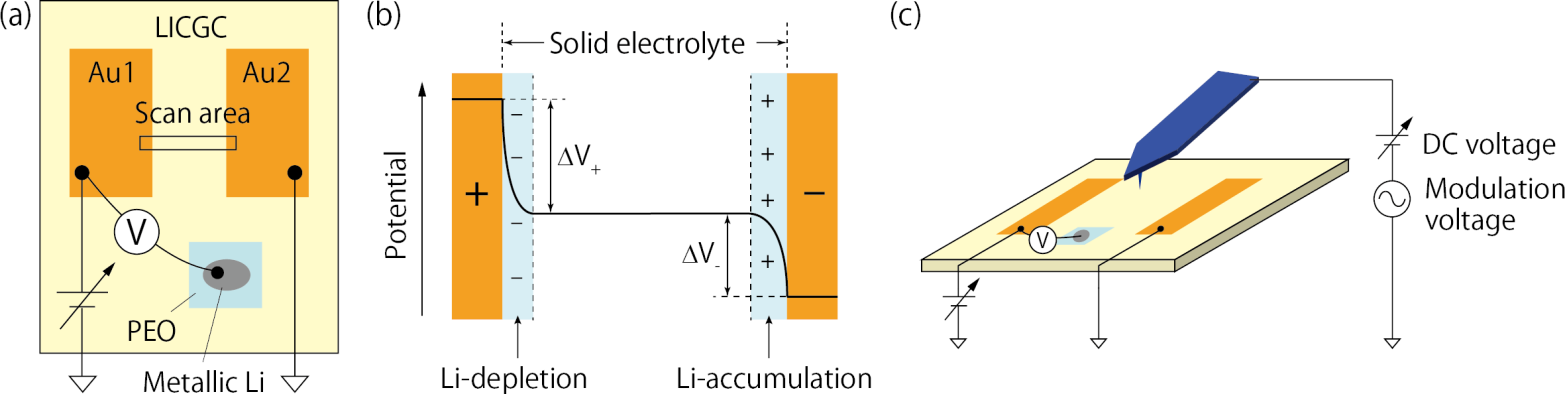
(a) Schematic diagram of the sample structure. Two Au electrodes and one metallic Li electrode are placed on the solid electrolyte sample (Li-ion conducting glass ceramic, LICGC^TM^). A poly(ethylene oxide)-based polymer electrolyte film, denoted PEO, is inserted between the metallic Li and the solid electrolyte to avoid reduction of Ti ions. A DC voltage is applied between the two Au electrodes. The left Au electrode (Au1) potential relative to the metallic Li electrode (vs Li^+^/Li) is measured using a voltmeter. The scanned area is indicated by the solid square. (b) Schematic illustration of internal potential distribution when a DC voltage is applied between the Au electrodes shown in (a). (c) Schematic illustration of experimental setup of KPFM measurement.

We begin with a general discussion of how the internal potential distribution changes when a DC voltage is applied between the electrodes in a simple electrochemical cell, as shown in Figure 1a. After applying the DC voltage for some time, the electric field in the solid electrolyte becomes shielded by the formation of a Li-depletion layer on the positive electrode side and a Li-accumulation layer on the negative electrode side [14–15]. Consequently, a voltage drop occurs only close to the interface between the electrode and the solid electrolyte, with the potential within the solid electrolyte region becoming constant, as depicted in Figure 1b. In general, the magnitude of the potential drop that occurs on the positive (ΔV_+_) and negative electrode sides (ΔV_−_) cannot be predicted using only the applied DC voltage. This makes it difficult to analyze the progression of redox reactions at the electrodes, because the redox reactions depend strongly on the potential difference at the electrode–electrolyte interface (ΔV_+_, ΔV_−_).

**Figure 2:**
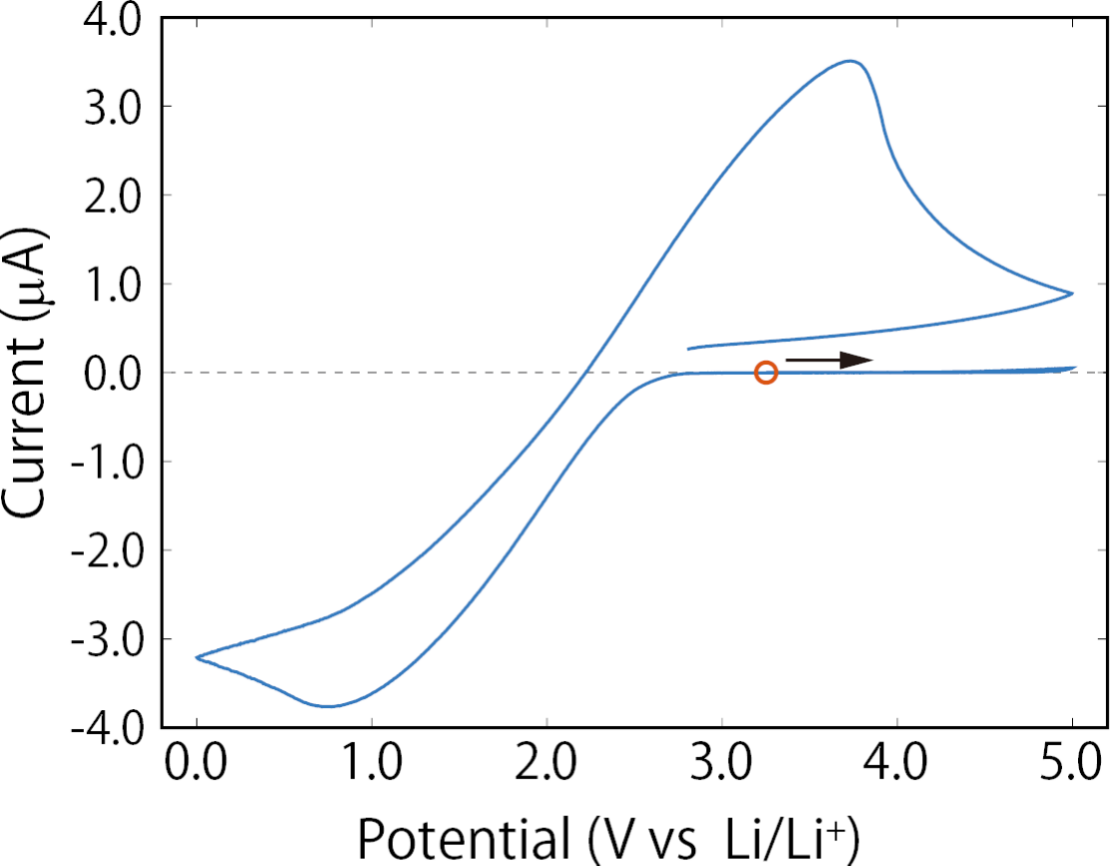
CV curve obtained by applying a voltage to the Au electrode deposited on the solid electrolyte with respect to the Li reference electrode. The scan rate was 0.5 mV/s, and the potential range was 0 to 5 V (vs Li/Li^+^). The potential sweep is initiated in the positive direction from the open-circuit voltage (3.2 V vs Li/Li^+^), indicated by an open circle.

Before explaining the results of the KPFM measurements, we discuss the electrochemical property of the Au electrode–solid electrolyte interface. Figure 2 shows a cyclic voltammetry (CV) curve obtained by applying a voltage to the Au electrode deposited on the solid electrolyte with respect to the Li reference electrode. The sample for the CV measurement was prepared separately from the sample used for the KPFM measurement. The potential range was 0 to 5 V (vs Li/Li^+^). Initially, the potential was set to open-circuit voltage (3.2 V vs Li/Li^+^) and swept in the positive direction. Large anodic and cathodic currents were observed at around 3.75 and 0.75 V, respectively. Considering their potential positions, the current can be attributed to the oxidation and reduction of Ti ions in the solid electrolyte [16]. The redox potential of Ti ions can be estimated to be about 2.3 V (vs Li/Li^+^). During the initial potential sweep from 3.2 to 5 V, the anodic current was negligibly small. This is because Ti ions around the Au electrode had not been reduced, thus prohibiting further oxidation of Ti ions. After that, when the potential was swept from 5 to 0 V in the negative direction, a cathodic current due to the reduction of Ti ions started to flow at around 2.8 V (vs Li/Li^+^).

**Figure 3:**
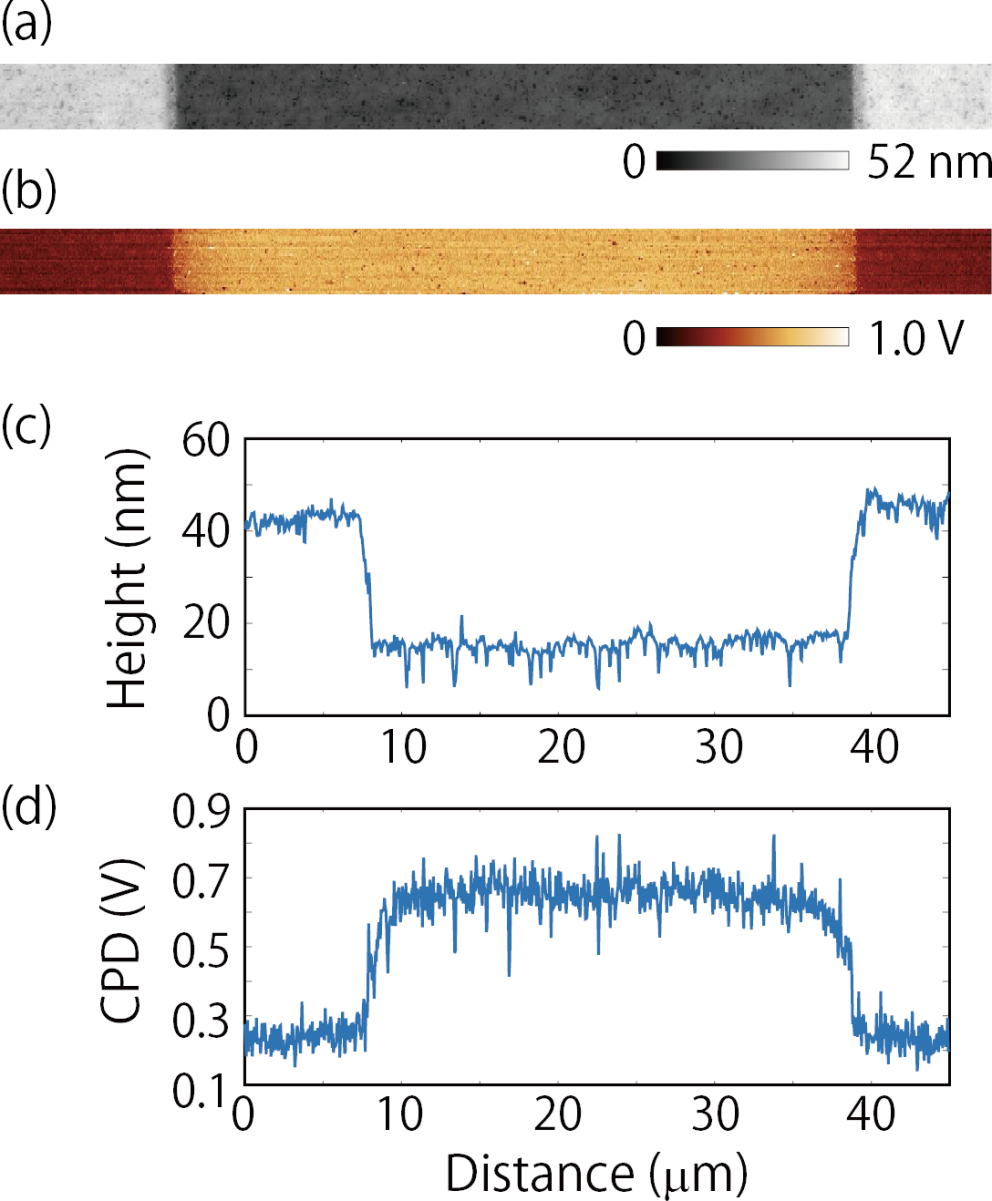
(a, b) Topography and CPD images, respectively, obtained across the solid electrolyte region (indicated in Figure 1a) when 0 V was applied between the Au electrodes. Image size is 45 μm × 3 μm. Scan rate is 0.1 Hz. (c, d) Cross sections of topography and CPD taken from the images in (a) and (b), respectively.

**Figure 4:**
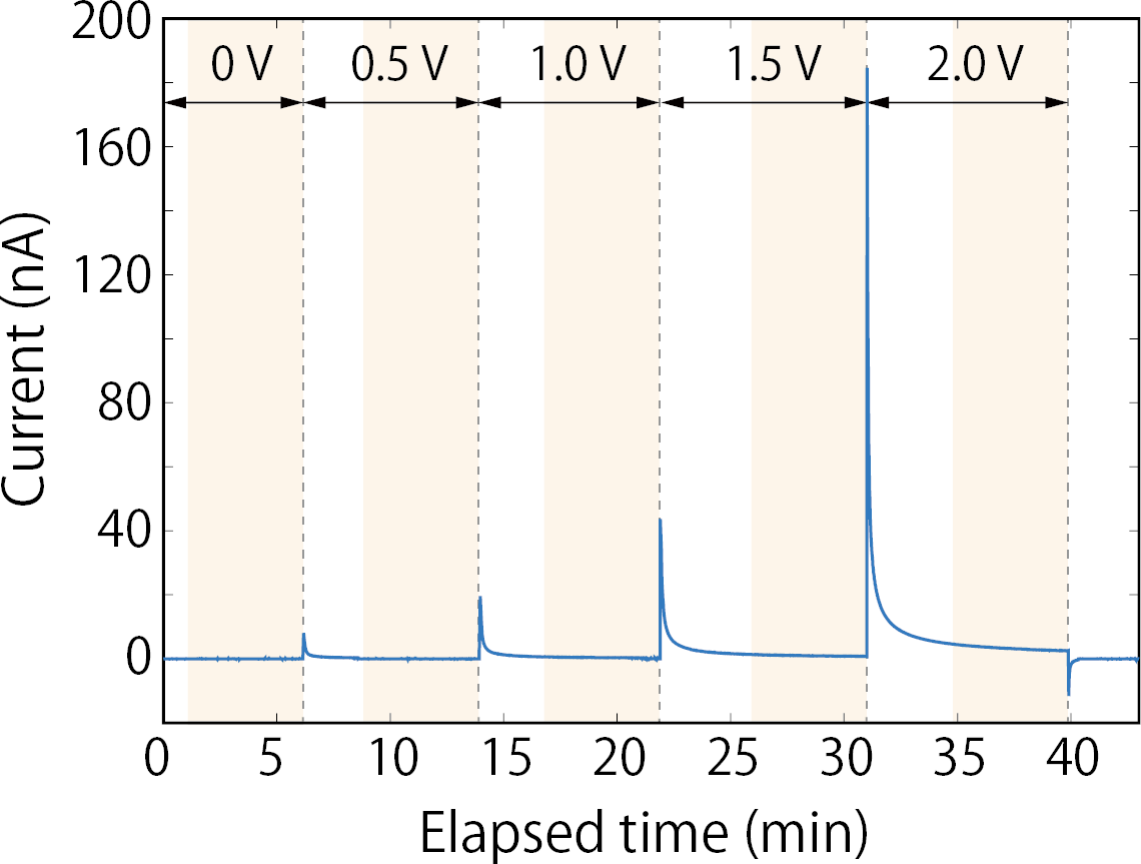
Current flowing between the Au electrodes during the forward bias sweep, plotted as a function of elapsed time. DC voltages applied between the Au electrodes are shown at the top of the graph. The periods during the KPFM measurements are shaded.

KPFM measurements were performed in the region across the solid electrolyte (Figure 1a). Figure 3a and Figure 3b display the topography and CPD images, respectively, obtained when 0 V was applied between the Au electrodes. The cross sections of the topography and CPD taken from the images in Figure 3a and Figure 3b are shown in Figure 3c and Figure 3d, respectively. The CPD values measured under this condition reflect the work-function difference across the surface [3]. The CPD values in the solid electrolyte region were about 0.4 V higher than those in the Au electrode regions, as depicted in Figure 3d. We then conducted KPFM measurements by varying the applied DC voltage from 0.5 to 2.0 V in 0.5 V increments. When the DC voltage is applied, the Li ions in the solid electrolyte move toward the negatively biased electrode, resulting in an ionic current flow. The ion current decays with time and, in principle, becomes zero when the electric field in the solid electrolyte is shielded by the accumulation and depletion of Li ions. Before starting each KPFM measurement, we waited 2–4 min until the current decayed sufficiently to near saturation. Figure 4 shows the current flowing between the Au electrodes during the forward bias sweep. The periods during the KPFM measurements are shaded. The finite currents were still flowing during the KPFM measurements, which can be attributed mainly to the electronic conduction. Importantly, even when we waited longer (more than 1 h), no apparent changes were observed in the potential distributions measured by the KPFM. This suggests that the KPFM measurements were performed under nearly equilibrium conditions.

**Figure 5:**
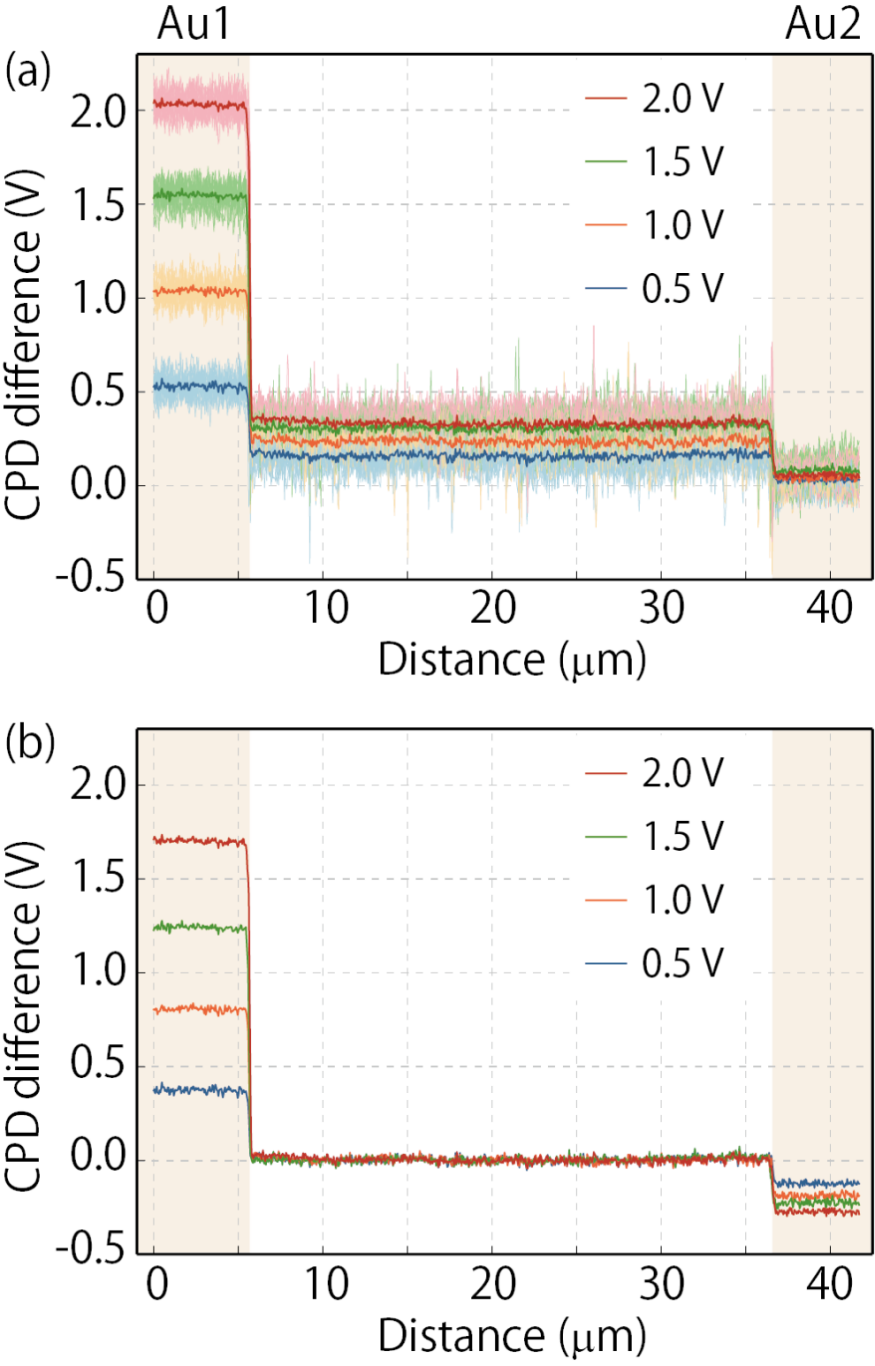
Line profiles of the CPD difference taken from the KPFM images obtained across the solid electrolyte region. (a) The CPD difference was calculated by subtracting the CPD values measured when 0 V was applied between the two Au electrodes from those at the same locations when a DC voltage varying between 0.5 and 2.0 V was applied. 15 profiles were taken from each image obtained with different applied DC voltages and plotted in light colors. Their average values are plotted in dark colors. (b) The line profiles shown in (a) are offset such that the potential of the electrolyte region becomes zero, to analyze the change in electrode potential relative to the potential in the solid electrolyte region. The regions of Au electrode are shaded.

To extract the changes in the internal potential distribution caused by the applied DC voltage [17,7,12], we subtracted the CPD values measured when 0 V was applied from those at the same locations when the DC voltage from 0.5 to 2.0 V was applied. The line profiles of the CPD difference (change in the surface potential) obtained by this subtraction are shown in Figure 5a. In all the data, a voltage drop occurs at the Au electrode–solid electrolyte interfaces, and the potential change in the solid electrolyte region is constant. These results are direct experimental evidence that the electric field in the solid electrolyte was shielded by the accumulation and depletion of Li ions. In this experiment, the Au2 electrode was connected to the ground; therefore, the CPD changes at the Au2 electrode were negligibly small, and the potentials at the Au1 electrode were almost the same as the applied voltage. As mentioned above, analyzing only the potential changes at the Au1 electrode relative to ground cannot reveal the potential difference across the electrode–solid electrolyte interface.

**Figure 6:**
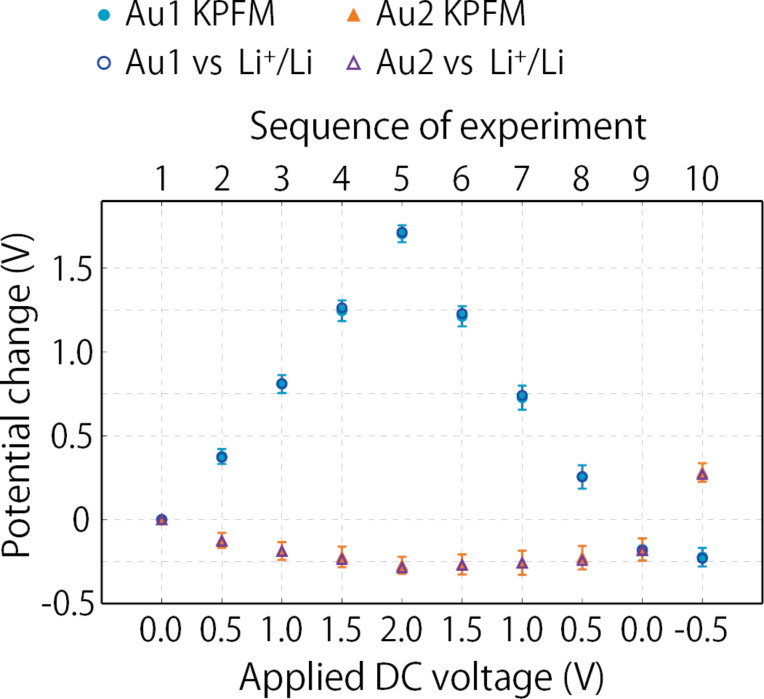
Changes in the Au1 and Au2 electrode potentials induced by applying a DC voltage between them. The DC voltage was varied from 0 to 2.0 V (forward sweep) and from 2.0 to −0.5 V (backward sweep) in steps of ±0.5 V. Solid circles and triangles represent the changes in surface potential relative to the surface potential in the solid electrolyte region, as extracted from the KPFM data. Open circles and triangles represent the changes in electrode potential (vs Li/Li^+^) measured using a voltmeter, shown in Figure 1.

To evaluate the change in surface potential distribution relative to the surface potential in the solid electrolyte region, we offset the line profiles shown in Figure 5a, such that the CPD difference over the solid electrolyte region became zero, as shown in Figure 5b. Note that the electrochemical properties of the solid electrolyte in the bulk have to remain unchanged during the KPFM measurements to justify the offset process. These line profiles highlight the fact that the applied DC voltage not only increased the Au1 electrode potential but also decreased the Au2 electrode potential. Figure 6 plots the changes in the surface potential at the Au1 (solid circles) and Au2 electrodes (solid triangles) relative to the surface potential in the solid electrolyte region for each applied DC voltage. This graph also includes the data acquired by changing the DC voltage from 2.0 to −0.5 V in −0.5 V decrements, just after acquiring the data shown in Figure 5.

During the forward voltage sweep and between 2.0 and 0.5 V in the backward sweep, the voltage drop across the Au1 electrode–solid electrolyte interface was greater than across the Au2 electrode–solid electrolyte interface. This asymmetric potential drop arises from the differences in the electrochemical properties of the two interfaces. As the DC voltage increased, the potential of the Au2 electrode decreased relative to that of the solid electrolyte region. When the Au2 electrode potential approached the reduction potential of Ti ions in the solid electrolyte, Ti ions near the Au2 electrode began to be reduced, which suppressed a further decrease in the electrode potential. As mentioned above, the reduction of Ti ions is expected to begin when the potential of Au2 electrode reaches about 2.8 V (vs Li/Li^+^). However, the Ti ions near the Au1 electrode remained unchanged because the Au1 electrode potential remained higher than the Ti reduction potential during the voltage sweep (see Table 1 where the Au1 and Au2 electrode potentials relative to the Li reference electrode during KPFM measurements, monitored by a voltmeter, are summarized). In contrast, when the polarity of the DC voltage was changed from 0 V to −0.5 V in the backward sweep, the change in the Au1 electrode potential was small because the Au1 electrode potential reached the Ti reduction potential, while the Au2 electrode potential varied significantly.

**Table 1:** Potential of Au1 and Au2 electrodes relative to the Li reference electrode at each applied DC voltage during the KPFM measurements.

Applied DC voltage (V)	Au1 (V vs Li/Li^+^)	Au2 (V vs Li/Li^+^)

0	2.978	2.978
0.5	3.350	2.850
1.0	3.790	2.790
1.5	4.241	2.741
2.0	4.692	2.692
1.5	4.207	2.707
1.0	3.719	2.719
0.5	3.235	2.735
0.0	2.794	2.794
−0.5	2.748	3.248

Figure 6 shows that, for a given applied DC voltage, the changes in surface potential varied depending on the history of the voltage application. For example, when the voltage was set to 0 V in the backward sweep, the potentials at both the Au1 and Au2 electrodes dropped by approximately 0.18 V relative to the initial state (0 V in the forward sweep). This is because the electrochemical properties at the Au electrode–solid electrolyte interface vary owing to the reduction of Ti ions. These results prove that, even if the DC voltage is accurately controlled, the electrode potential depends on the electrochemical properties of the interfaces, and that simply measuring the potential relative to ground using KPFM is not sufficient to analyze the electrochemical system.

We verified the hypothesis that the changes in surface potential relative to the surface potential of the solid electrolyte (Figure 6) were equivalent to the changes in the electrode potential as measured by standard electrochemical measurements. For that, we compared the changes in surface potential with the changes in the electrode potential measured relative to the Li reference electrode shown in Table 1. Initially, the potential of both Au electrodes was 2.978 V (vs Li/Li^+^), higher than the reduction potential of Ti ions. In Figure 6, the changes in the Au1 and Au2 electrode potentials with respect to the initial electrode potential (2.978 V) are plotted as open circles and open triangles, respectively. The changes in the electrode potential agree well with those obtained using KFPM. This demonstrates that the potential in the solid electrolyte region can be utilized as a stable reference in KPFM measurements, similar to standard electrochemical measurements.

## Conclusion

We verified the possibility of using a stable potential reference in KPFM measurements without attaching a reference electrode to the sample. For this purpose, we conducted KPFM measurements on a simple three-electrode electrochemical cell, where two Au electrodes and one metallic Li reference electrode were placed on the solid electrolyte substrate. The changes in the surface potential of the Au electrodes, measured relative to the surface potential of the solid electrolyte region, agreed well with the changes in the Au electrode potential measured relative to the metallic Li electrode using a voltmeter. These results suggest that the solid electrolyte region could be used as a potential reference as long as the electrochemical properties of the solid electrolyte in the bulk do not change during the KPFM measurements due to electrochemical reactions or other reasons. These findings provide the foundation for future work on the analysis of KPFM data derived from electrochemical devices, enabling the thorough characterization of redox reactions occurring at electrode–electrolyte interfaces.

## Experimental

### KPFM measurement

The KPFM measurements were performed at room temperature using a commercial atomic force microscope (Park Systems, NX10) placed in an Ar flow glove box (O_2_: *<*1 ppm, H_2_O: *<*1 ppm). We used Cr/Pt-coated Si cantilevers (Budget Sensors, Multi75E-G) with a nominal resonance frequency of 75 kHz and a spring constant of 3 N/m. The CPD was detected using the sideband KPFM mode [18–19,4]. The amplitude and frequency of the modulation voltage were 1.5 V and 3.2 kHz, respectively. The modulation voltage and DC voltage were applied to the tip to minimize the electrostatic force between the tip and sample, as shown in Figure 1c. An electrometer (Keithley 617) was used to measure the Au electrode potential relative to the Li reference electrode. A DC voltage was applied between the Au electrodes and the resulting current was measured using an electrometer (ADCMT 8252).

### Sample preparation

The solid electrolyte sample was a Li-ion conducting glass ceramic purchased from OHARA Inc. (LICGC^TM^ AG-01) [20]. The size and thickness of the substrate were 25.4 mm × 25.4 mm and 150 μm, respectively. The main crystalline phase was Li_1+_*_x_*_+_*_y_*Al*_x_*(Ti,Ge)_2−_*_x_*Si*_y_*P_3−_*_y_*O_12_ [20]. The substrate was cut into pieces of approximately 10 mm × 20 mm, and ultrasonically cleaned in ethanol. Subsequently, a 30 nm thick Au electrode was deposited by resistance-heating evaporation using a crucible in a vacuum chamber. A polyurethane-coated Cu wire (25 μm diameter) was used as a mask to pattern the electrode shapes, as shown in Figure 1a. We used narrow electrodes (≈100 μm) to suppress the non-local capacitive coupling between the electrode and the tip, which is expected to reduce the tip-averaging effect [21–23]. A metallic Li foil (Honjo metal Co., Ltd.) was used to measure the Au electrode potential versus Li/Li^+^. To avoid the reduction of Ti ions in the solid electrolyte owing to direct contact between the Li metal and solid electrolyte, a poly(ethylene oxide)-based polymer electrolyte film (Osaka Soda Co., Ltd.), denoted PEO, was inserted between them. Before the KPFM measurements, the sample was heated at 150 °C for 30 min using a hot plate in the glove box to remove a water layer that might be present on the surface.
